# Economic hardship among principal family caregivers of cancer patients at Khartoum oncology hospital 2020: a cross-sectional study

**DOI:** 10.1186/s12913-022-08918-y

**Published:** 2022-12-08

**Authors:** Ammar Elgadi, Aseel Hisham, Hayat A. Ahmed, Hiba Ali Elzaki, Kamil Merghani Ali shaaban, Ola Dafaalla, Osama Ahmed Elkhidir, Salma S. Alrawa, Tahani Amin Mahmoud, Waad Wadidi

**Affiliations:** 1 Faculty of Medicine, University of Khartoum, Khartoum, Sudan; 2 Assistant professor community medicine department National University, Khartoum, Sudan; 3 Associate professor community medicine department University of Khartoum, Khartoum, Sudan; 4 Assistant Professor National University, Khartoum, Sudan

**Keywords:** Economics, Cancer, Caregivers, Sudan, Oncology

## Abstract

**Background:**

The impact of cancer extends beyond patients and consumes their families. Family members are widely recognized as informal caregivers. The economic burden on family caregivers is increased with new treatments, prolonged survival, and reduced stay in the acute care setting. This is especially true in African countries where family bonds are sacred and health system is fragile that they need to pay out of pocket for care.

The aim of this study is to estimate the perceived caregivers’ economic burden in the subsequent aspects: financial strain, inability to make ends meet, not enough money for necessities, and economic adjustments/cutbacks.

**Method:**

This study was a quantitative, descriptive cross-sectional study conducted at Khartoum oncology hospital. Included 143 caregivers of cancer patients. Data were collected through face-to-face interviews using the socio-demographic Questionnaire and Economic Hardship Questionnaire (EHQ).

**Results:**

One hundred forty-three cancer patients and their caregivers were included. 56.6% of patients were females, and about 32.2% were aged 51–65 years. The most common cancer types were breast cancer and leukemia. Roughly 33% of patients had stage IV cancer on presentation, and about 53.9% received chemotherapy. Unlike cancer patients, (47.6%) of family caregivers were aged 18–34 years, yet they were mainly females (54.4%). Most of them (34.3%) were unemployed, with a mean monthly gross income of 53.3 dollars, while the mean household monthly gross income was 113.0 dollars. The mean score of the economic hardship scale was 35.8 out of 64. Most of the caregivers experience no difficulties affording necessities. However, they experience difficulties with medical and leisure activities.

There was no significant association between caregiver economic hardship and cancer patient characteristics (patients' age, cancer stage, and treatment type). However, there was a significant association between caregivers' economic hardships and their gender, marital status, educational level, occupation, caregiver monthly gross income, and household monthly gross income.

**Conclusion:**

The study findings suggest a moderate financial burden among cancer caregivers. The predicting factors include being single, a student, male, of higher educational level, and lower income. Financial difficulties are associated with maladaptive behavior and should come to light.

## Background

Cancer is a global health problem and one of the most common causes of death [1]. Sudan is no exception, with malignant disorders contributing to about 50,000 deaths annually, and the number is expected to increase [2].The impact of cancer extends beyond patients and consumes their family members since they are widely recognized as informal caregivers [3, 4]. The economic burden on family caregivers is increased with new treatments, prolonged survival, and reduced stay in an acute care setting [5]. This is especially true in African countries where family bonds are sacred, and health systems are fragile that they are deeply burdened by the disease [6].

The economic burden entails both direct and indirect costs. Direct costs include treatment and other expenses inside the healthcare facility, as well as at-home nursing care when a patient has difficulty functioning. Indirect costs include transportation fees and partial or total loss of livelihood to provide adequate medical measures at home [7]. In the USA, it is estimated that caregiving accounts for 18–33% of total cancer costs, mostly due to hours spent in providing care [8]. Indeed, financial hardship hits caregivers along with cancer patients. As the financial burden increases, treatment compliance is disturbed, leading to a vicious cycle of more systemic manifestations, more days in acute care, and more spending [9].

An international panel of experts sets priorities for cancer caregiver research to influence practice, education, and policy. The financial impact of caregiving was agreed upon as an area of research by consensus [10].

Sudan has a low health expenditure, its healthcare system is affected by the country’s economic instability and despite the efforts of the National Cancer Department in the Federal Ministry of Health regarding supporting the oncology centers in term of providing some free medications in chemotherapy and radiotherapy, and there is a huge gap in financing those services. Families and principal caregivers mainly cover this finance gap [11]. Up to authors’ knowledge, there is a limited studies in cancer economics burden particularly on caregiver.

### Objectives and aims

The aim of this study is to estimate the perceived caregivers’ economic burden in the subsequent aspects: financial strain, inability to make ends meet, not enough money for necessities, and economic adjustments/cutbacks. Additionally, to assess the association between economic burden and characteristics of principal family caregiver and patient.

## Methods

### Study design and area

A quantitative, descriptive, cross-sectional facility-based study was conducted at Khartoum Oncology Hospital, Khartoum State. In the year 2020. It was founded in 1967 and serves 80% of Sudan cancer patients. Therefore, it could be representative of cancer patients in the country overall [12].

### Study participants

The study included 143 caregivers of cancer patients diagnosed 3 months earlier from data collection. Participants were selected by systematic random sampling technique. Using the following formula:

$$n=\frac{N{z}^{2}\left(1-p\right)}{N{d}^{2}p+{z}^{2}p(1-p)}$$

where:

*N* ≡ Total number of registered cancer patients **33,201**

z ≡ Z value (i.e., 1.96 for 95% confidence level)

*p* ≡ Fraction of phenomena of interest (cancer prevalence) [12] **0.007**

*d* ≡ Desired margin of error, expressed as decimal **(0.05)**

*n* ≡ Sample size

Systematic random sample was implemented to recruit participants. Records from Khartoum Oncology Hospital were used as a sample frame to recognize patients with established diagnosis of cancer.

### Data collection method

A face-to-face structed interview was conducted after obtaining written informed consent. Data was collected using Economic Hardship Questionnaire (EHQ). The medical files were used to obtain clinical information.

Economic Hardship Questionnaire is a tool which was structured through the effort of Manuel Barrera and other in 2001. It provides a consideration of coherent reflection of perceived economic hardship [12].Contributors valued their psychological sense of economic burden in the subsequent aspects: financial strain, inability to make ends meet, not enough money for necessities, and economic adjustments/cutbacks. Individual items were calculated to produce an overall score for each subscale, with higher scores indicating higher levels of economic hardship [3].The over-all score of EHQ is 64 and greater scores indicate greater perceived sense of economic hardship.

The reliability of the EHQ was checked by Manuel Barrera to find that, The Kuder–Richardson reliabilities for economic Adjustments and Cutbacks scale were 0.70 and 0.73 for mothers and fathers, respectively. Internal consistency reliabilities (Cronbach’s alpha) for Not Enough Money for Necessities were 0.85 and 0.88 for mothers and fathers, respectively. The Cronbach alpha for Inability to Make Ends Meet was 0.77 for mothers and 0.71 for fathers. The Internal consistency reliabilities between the two items of financial constrain was 0.74 for mothers and 0.72 for fathers [12].EHQ was translated to Arabic language by the authors, checked by health economist, statistician, and epidemiologist. A pilot study was conducted to check the tools validity.

Economic Hardship Questionnaire reflects the perceived economic hardships. The overall score of (EHQ) is 64; greater scores indicate a greater perceived sense of economic hardship.

The medical files were used to obtain clinical information.

### Data management and analysis

Questionnaires and records were refined and managed carefully. Data were cross- checked for duplication, inaccurate entries, and completeness. Statistical Package for the Social Sciences (SPSS) ® version 20 was used for data entry and statistical analysis. Socio-demographic and medical information were presented in Percentages, frequencies, means and standard deviations. ANOVA and Chi square test of independence were applied to examine relationships among demographic, medical, social, and economic characteristics- with caregiver-economic hardship.

## Results

### Cancer patients & family caregivers’ basic characteristics

A total of 143 cancer patients and their caregivers were included in the study. About 32.2% were in the age group (51–65) while only 6.3% were (below 18). The mean age of cancer patients was 49.1(SD = 19.2). Females constitute 56.6% of patients. Most common cancer types were breast cancer (11.9%), leukemia (11.9%), lymphoma (9.8%), endometrial cancer (8.4%), and ovarian cancer (7.7%). About 32.9% of patients presented at stage IV of cancer, 22.9% at stage I, 22.9% at stage III while only 21.4% presented at stage II. Regarding treatment modalities, 53.9% patients were treated by chemotherapy, 16.3% by Chemotherapy plus Surgery and 19.9% were treated with other treatment modalities including radiotherapy.

Unlike cancer patients, (47.6%) of family caregivers were in the age group of (18–34), the mean age was 37.7 (SD = 13.2), yet they were mainly females (54.4%). Only (35.7%) of caregivers were original residents at Khartoum State.

### Economic characteristics of caregivers

Little more than half of patients had health insurance (53.1%). It was mostly social (93.5%), and the rest were employment scheme (6.5%). None of the patients had private insurance. The insurance coverage of cost is mostly partial (80.3). Only (7.9%) of contracts covered the whole cost and (11.8%) does not include cancer in the insurance.

Most of the caregivers (34.3%) were unemployed. Among employed caregivers 54.3% were on leave as a consequence of caregiving. The mean caregiver monthly gross income was 2540 SDGs (53.3 dollars), while the mean of household monthly gross income was 5379 SDGs (113.0 dollar). Table1.

**Table 1:** Demographic and economic characteristics of family caregiver of cancer patients, Khartoum Oncology Hospital

Variable	Category	Count (%)
**Age**	18–34	68 (47.6)
	36–50	48 (33.6)
	51–65	24 (16.8)
	> 65	3 (2.1)
**Gender**	Male	65 (45.5)
	Female	78 (54.5)
**Occupation**	Officer	18 (13.1)
	Farmer	12 (8.8)
	Merchant	13 (9.5)
	Free worker	44 (32.1)
	Household	47 (34.3)
	Student	3 (2.2)
**Current employment** **status**	Full Time	28 (29.8)
	Part timer	14 (14.9)
	On leave	51 (54.3)
	Retired	1 (1.1)
**Caregivers monthly gross income**	0–450	50 (35.0)
	451–1500	40 (28.0)
	1501–3000	30 (21.0)
	3001–5000	13 (9.1)
	5001–10,000	7 (4.9)
	> 10,000	3 (2.1)
**Household monthly gross income**	0–450	9 (6.3)
	451–1500	29 (20.3)
	1501–3000	36 (25.2)
	3001–5000	27 (18.9)
	5001–10,000	30 (21.0)
	> 10,000	12 (8.4)

### Family caregiver perceived economic hardship

When evaluating financial strains using Economic Hardship Questionnaire (EHQ), 26.6% of caregivers experienced bad times once in a while, and 28.7% indicate that they sometimes lack basic needs.

With regard to inability to earn enough money among family caregivers, 10% of them expected to have a great deal of difficulty with paying bills; but 28.0% expected no difficulty of any kind. A percentage of 14.7% expected to have a great deal of financial difficulty at the end of each month; then again 17.5% expected no difficulty at all, Table 2.

**Table 2:** financial strains and social characteristics of family caregiver of cancer patients

Item	Category	Count (%)
**Experience bad times**	Almost never	27 (18.9)
	Once in a while	38 (26.6)
	Sometimes	35 (25.5)
	Often	36 (25.2)
	Almost always	7 (4.9)
**Lack of basic things**	Almost never	32 (22.4)
	Once in a while	31 (21.7)
	Sometimes	41 (28.7)
	A lot of the time	30 (21.0)
	Almost always	9 (6.3)
**Difficulty with paying bills**	Considerable difficulty	15 (10.5)
	Quite a bit of difficulty	27 (18.9)
	Some difficulty	30 (21.0)
	A little difficulty	31 (21.7)
	No difficulty at all	40 (28.0)
**End up with at the end of month**	Considerable difficulty	21 (14.7)
	Quite a bit of difficulty	27 (18.9)
	Some difficulty	29 (20.3)
	A little difficulty	41 (28.7)
	No difficulty at all	25 (17.5)

Most of caregivers experience no difficulties to afford necessities such as clothing, household appliances, transport, and food. However, they experience difficulties with medical and leisure activities Table 3.

**Table 3:** Affordability of necessities among family caregivers of cancer patients

Item	Disagree	Neutral	Agree
	**Count (%)**	**Count (%)**	**Count (%)**
**Affordability of suitable home**	47 (32.9)	11 (7.7)	85 (59.5)
**Affordability of suitable clothing**	51 (35.7)	15 (10.5)	77 (53.9)
**Affordability of suitable household appliances**	61 (42.7)	11 (7.7)	71 (49.7)
**Affordability of suitable transport**	53 (37.1)	11 (7.7)	79 (55.3)
**Affordability of suitable food**	19 (13.3)	26 (18.2)	98 (68.6)
**Affordability of suitable medical care**	68 (47.6)	15 (10.5)	60 (42.0)
**Affordability of suitable leisure**	116 (81.1)	9 (6.3)	18 (12.6)

With reference to economic adjustments, 58.7% of family caregivers made considerable changes to their food shopping or eating habits to save money, 72.7% asked relatives or friends for money to get by, and 53.1% sold some possessions. The mean score of the economic hardship scale was 35.8 out of 64. Table 4.

**Table 4:** Economic adjustments/cutbacks among family caregivers of cancer patients

Item	Yes	No
	**Count (%)**	**Count (%)**
**Change shopping or eating habits to save money**	84 (58.7)	59 (41.3)
**Shut down electrical instruments to save money**	49 (34.3)	94 (65.7)
**Not seeing a doctor when necessary**	40 (28.0)	103 (72.0)
**Difficulty paying bills**	61 (42.7)	82 (57.3)
**Asked relatives or friend for money**	104 (72.7)	39 (27.3)
**Added another job or change current one**	22 (15.4)	121 (84.6)
**Received financial assistance**	4 (2.8)	139 (97.2
**Sold some possessions**	76 (53.1)	67 (46.9)
**Moved house to save some money**	6 (4.2)	137 (95.8)

### Associations between cancer patient’s/ caregiver characteristics and economic hardship

ANOVA tests revealed no statistical association between caregiver economic hardship and cancer patient`s characteristics (patients’ age, cancer stage and treatment type) Table 5.

**Table 5:** Association between caregiver economic hardship and cancer patient’s characteristics:

**Item**	**Category**	**Count**	**Economic hardship score**		test statistic^*^	***P*-value**
			**Mean**	**SD**		
**Patient age**	0–17	9	34.4	5.0	0.6759	0.610
	18–34	25	35.0	6.3		
	35–50	36	35.4	5.9		
	51–65	46	36.8	5.5		
	> 65	27	36.1	5.31		
**Cancer stage**	Stage I	32	36.1	5.5	0.5656	0.666
	Stage II	30	36.6	4.9		
	Stage III	32	34.8	5.9		
	Stage IV	46	35.8	6.0		
**Treatment type**	Chemotherapy	76	35.7	5.7	2.8537	0.643
	Radiotherapy	6	35.3	6.9		
	Surgery	2	28.5	0.7		
	Other	21	36.0	6.2		
	Chemotherapy + Surgery	23	36.6	5.5		
	Chemotherapy + Radiotherapy + Surgery	6	36.3	4.7		

^*^ One way ANOVA F value

Student’s t test revealed association between caregiver economic hardships and their gender (*p* = 0.016). ANOVA test revealed association between caregiver economic hardships and marital status (*P* = 0.001), educational level (*P* = 0.006), occupation (*P* = 0.019), caregiver monthly gross income (*P* = 0.001), and household monthly gross income (*P* < 0.001), Table 6.

**Table 6:** Association between caregiver economic hardship and caregiver’s characteristics:

**Item**	**Category**		**Economic hardship Score**		**test statistic**	***P*-value**
		**count**	**Mean**	**SD**		
**Gender**	Male	65	36.1	5.1	0.5253^**^	0.6002
	Female	78	35.6	6.1		
**Marital status**	Single	42	37.6	5.6	0.7637^*^	0.001
	Married	99	35.8	5.4		
	Divorced	1	31.0	5.1		
	Widowed	10	29.9	4.0		
**Educational level**	Illiterate	29	33.0	6.2	5.8258^*^	0.006
	Khalwa	11	33.4	2.5		
	Primary	30	36.0	5.6		
	Secondary	42	36.6	5.3		
	University	7	39.9	4.2		
	Postgraduate	24	37.5	5.6		
**Occupation**	Officer	18	37.4	4.3	2.8537^*^	0.019
	Farmer	12	32.5	6.5		
	Merchant	13	38.9	6.7		
	Free worker	44	34.6	5.0		
	Household	47	36.4	5.5		
	Student	3	39.3	4.7		
**Caregiver monthly gross income**	0–450	50	36.6	5.5	4.6624^*^	0.001
	451–1500	40	33.8	4.9		
	1501–3000	30	34.2	5.3		
	3001–5000	13	39.5	5.9		
	5001–10,000	7	39.6	6.2		
	> 10,000	3	42.0	2.7		
**household monthly gross income**	0–450	9	34.1	5.3	5.6993^*^	< 0.001
	451–1500	29	32.9	4.0		
	1501–3000	36	34.8	5.7		
	3001–5000	27	36.5	5.8		
	5001–10,000	30	37.7	5.1		
	> 10,000	12	41.2	5.2		

^*^ One way ANOVA F value

^**^ Student t test t value

## Discussion

This is one of the first studies to examine the financial burden of cancer among Sudanese cancer caregivers. More than half of cancer patients (56.5%) were females mostly from the middle age group which is consistent with cancer surveys in Sudan [2]. The most prevalent cancers were breast cancer and leukemia and the overall distribution of cancer types resembled that of cancer registry with minimal exceptions [2].

Most patients presented at a late stage which is common in developing countries [3, 13, 14]. In this study -as in literature- the stage is not related to financial burden [3].

Family caregivers were predominantly females. The caregiving role of females is recognized in many studies across the globe [10, 14].This may be explained by the general culture of leaving caregiving for females and financial support for males [15]. It may also reflect the epidemiology of cancer; as breast cancer was the most prevalent and societal norms prefer females in caring for this specific cancer, the second is leukemia and mothers’ caregiving role is globally recognized [16]. The mean age of caregivers is younger than in global literature [17, 18]. A similar age is reported from Uganda [19] and other countries of Sub-Saharan Africa [15] matching the youth of these countries.

As most caregivers were females, it was not strange that they were primarily unemployed (household). Among those who had jobs, more than half were on leave, which is a recognized incident that exacerbates the financial burden of cancer [20].

It was remarkable that most caregivers reported household monthly income that does not exceed 120 dollars which reflects a low socioeconomic status. This is understandable as more wealthy families are likely to get care in private centers or outside Sudan. Over half of the participants had health insurance. In spite of this caregivers reported experiencing bad times more than once in variable frequencies resulting in lack of basic things as an adaptive behavior. Similarly, they had difficulties in paying bills. These difficulties are consistent in developed and developing countries [3, 21, 22].Family caregivers went through average economic hardship as reflected by economic hardship scale (35.8 out of 64).

The main adapting behaviors were borrowing money, selling some possessions, and cutting down eating and shopping reservations as well as postponing medical care visits. Postponing medical care is alarming due to the detrimental effect of non-adherence and the vicious cycle it induces [9].

The study revealed that being single, student, male, of higher educational level and lower income was proportional to higher perceived economic hardship. The association between lower income and economic burden is established in literature [23]. In contrary, other studies found that being female and of lower educational level are associated with higher perceived burden [23]. The association between gender, education and perceived financial stress should be investigated. A possible explanation in our case is the concept of contentedness. Those of lower educational may accept the situation as it is without further analysis.

We may argue that in Sudan educated males calculate the expenses thus report higher burden while females and uneducated tend to accept the situation as it is.

Patient age, cancer stage and treatment type were not significantly associated with economic burden of caregivers in our study and other studies [3]. It is noteworthy that they are associated with economic burden perceived by cancer survivors themselves [22].

This study is important as it addresses the important but neglected issue of financial difficulties among caregivers of cancer patients in one of low-income countries. It was conducted in the main tertiary oncology center in Sudan that covers 80% of the population so it reflects the general situation.

The study should be viewed in the light of some limitations; namely the reliability of using standardized tools in Sudan setting in terms of selected variables, diagnostic criteria and cut offs. Another limitation is the pure use of quantitative approach which is insufficient to get to the depth of the problem. The general limitations of cross-sectional studies in validating associations should also be considered.

## Conclusion

The study findings suggest a moderate financial burden among cancer caregivers. The predicting factors include being single, student, male, of higher educational level and lower income. Financial difficulties are associated with maladaptive behavior, and they should come to light. We recommend that future studies use a qualitative approach to get into the depth of the problem and consider solutions suggested by caregivers themselves. Oncologists should also be included to assess their awareness of this problem and the methods they use in going through it. Meanwhile, oncologists are encouraged to screen for financial difficulties and review modifying factors with cancer patients and their caregivers.

## Data Availability

All data supporting the findings of this article will immediately be available upon request from corresponding author.

## Abbreviations

EHQ: Economic hardship questionnaire
SDG: Sudanese pound
SPSS: Statistical package for social sciences
